# What role does health literacy play in patients' involvement in medical decision-making?

**DOI:** 10.1371/journal.pone.0173316

**Published:** 2017-03-03

**Authors:** Anne E. M. Brabers, Jany J. D. J. M. Rademakers, Peter P. Groenewegen, Liset van Dijk, Judith D. de Jong

**Affiliations:** 1 NIVEL, the Netherlands Institute for Health Services Research, Utrecht, the Netherlands; 2 Department of General Practice, CAPHRI, School for Public Health and Primary Care, Maastricht University, Maastricht, the Netherlands; 3 Department of Sociology, Department of Human Geography, Utrecht University, Utrecht, the Netherlands; Western Sydney University, AUSTRALIA

## Abstract

Patients vary in their preferences towards involvement in medical decision-making. Previous research, however, gives no clear explanation for this observed variation in their involvement. One possible explanation might be health literacy. Health literacy refers to personal characteristics and social resources needed for people to access, understand and use information to make decisions about their health. This study aimed to examine the relationship between health literacy and self-reported patient involvement. With respect to health literacy, we focused on those competences relevant for medical decision-making. We hypothesized that people with higher health literacy report that they are more involved in medical decision-making. A structured questionnaire was sent to members of the Dutch Health Care Consumer Panel in May 2015 (response 46%, N = 974). Health literacy was measured using five scales of the Health Literacy Questionnaire. A regression model was used to estimate the relationship between health literacy and self-reported involvement. In general, our results did not show a relationship between health literacy and self-reported involvement. We did find a positive significant association between the health literacy scale appraisal of health information and self-reported involvement. Our hypothesis was partly confirmed. The results from this study suggest that higher order competences, that is to say critical health literacy, in particular, are important in reporting involvement in medical decision-making. Future research is recommended to unravel further the relationship between health literacy and patient involvement in order to gain insight into whether health literacy might be an asset to enhance patient participation in medical decision-making.

## Introduction

Patients are increasingly expected to be in charge of their health and to be involved in decisions about their health [1–3]. This is partly because of the recognition of the ethical imperative to involve patients properly in decision-making about their health, and also due to the growing evidence that patient participation has several benefits [2 4–6]. Examples of these benefits are increased patient knowledge, increased patient satisfaction with treatment decisions, reduced patient anxiety and better treatment adherence [5–8]. Furthermore, providing care that is respectful of, and responsive to, an individual patient’s preferences, needs and values is regarded as one of the key elements of good quality of care [9].

Several studies examined patients’ preferences towards involvement in medical decision-making. These studies observed that although some patient groups prefer to leave the decision to their physician, most patients want to share decisions with their physician [10–13]. Whether patients want to participate in medical decision-making is associated with a variety of factors. These include, among others: their relationship with health professionals; the type of decision they need to make; their experience of illness and medical care; their diagnosis and health status; and demographic characteristics, such as gender, age and level of education [13]. In general, women, younger people, and higher educated people prefer a more active role in decision-making [10 12 13]. Several studies also observed that patients largely experience their preferred style of decision-making [14–17].

The above-mentioned studies indicate that there is a variation in patients’ preferences towards an active role in medical decision-making. However, research so far is primarily descriptive and does not give a clear explanation for the variation observed in involvement in medical decision-making. A possible explanation is that the variation observed in involvement reflects differences in the ability of people to become an active participant with regard to their health, and the competencies they have acquired to make informed decisions about their health. An important theoretical concept in this context is “health literacy”. Health literacy has been considered as important determinant of health [18 19]. It is defined as “personal characteristics and social resources needed for people to access, understand and use information to make decisions about health” [20 21]. It refers to a resource that is integrated into people’s daily lives and includes information and decision-making skills that are necessary in a range of different contexts [22]. The focus has shifted, over time, from functional literacy, for example, reading health information, to a broader focus on higher order competences [23]. In this respect, Nutbeam’s definition of health literacy became influential. Nutbeam (2000; 2008) discerned three different sequential types of health literacy. These include functional literacy, that is a basic knowledge of reading and writing. Communicative or interactive literacy, involving more advanced cognitive and literacy skills which can be used in everyday activities and to apply new information to changing circumstances. And critical literacy, the most advanced cognitive skills which can be applied to critically analyze information and to use it to exert greater control over life events and situations [24 25]. Higher levels of health literacy enable people to engage in a range of actions aimed at enhancing health [25].

With regard to patient involvement in medical decision-making, it can be hypothesized that a high level of health literacy enables people to play a more active role. They are presumably able to obtain, understand, appraise and apply information required for medical decision-making better. The following phases can be discerned within medical decision-making: information exchange; deliberation; and deciding on which treatment to implement [26–29]. If both physician and patient are involved, they collect and share information with each other in the first phase, for example about possible treatment options. The second phase refers to the process of expressing and discussing treatment preferences. In the last phase, they decide together on the treatment [27]. Health competences such as finding and having enough information, understanding and appraising this information, and being able to engage with physicians are thus especially relevant in order for people to play an active role.

Several studies have already examined the relationship between health literacy and medical decision-making. In general, these studies observed that people with low health literacy desire less participation [30–36]. Most of the studies conducted, however, are based on specific patient groups, and only included functional health literacy such as reading instead of a broader concept of health literacy.

We aimed in this study to examine the relationship between health literacy and patient involvement in medical decision-making. We performed our study among a sample of health care users in the Netherlands, and focused on their self-reported involvement in medical decision-making. With respect to health literacy, we focused on those competences necessary for medical decision-making. These are: finding and having enough information; understanding and appraising the information; and the ability to engage with health care providers. As explained above, we hypothesized that people with higher health literacy report that they are more involved in medical decision-making.

## Materials and methods

### Setting

Data were collected from the Dutch Health Care Consumer Panel, which aims to measure opinions on, and knowledge of, health care as well as the expectations of, and experiences with, health care among a cross-section of the Dutch population (see for more detailed information [37]). The Dutch Health Care Consumer Panel is a so-called access panel. An access panel consists of a large number of people who have agreed to answer questions on a regular basis. Many background characteristics of the panel members are known such as their age, gender and highest level of education completed. At the time of this study (May 2015), the Consumer Panel consisted of about 12,000 people aged 18 years and older. Each individual panel member receives a questionnaire about three times a year and can resign from the panel at any time. At the start of their membership, panel members can choose whether they want to receive a postal or web-based questionnaire. There is no possibility of people signing up for the panel on their own initiative. The Dutch Health Care Consumer Panel is renewed on a regular basis. We recruit possible new members by means of two ways. First, we buy an address file from an address supplier. As such, possible new members for the panel are sampled at random from the general population in the Netherlands. Second, we recruit possible new members via general practices participating in the NIVEL Primary Care Database [38]. Data are processed anonymously and the protection of the data collected is registered with the Dutch Data Protection Authority (nr. 1262949). A privacy regulation is available for the Consumer Panel. There is no legal requirement to obtain informed consent nor approval by a medical ethics committee when conducting research through the panel [39]. For this study, a questionnaire was sent to a sample of 2,116 panel members in late May 2015. The sample consisted of all migrants (both western and non-western) included in the panel (N = 1,058) and the same number of non-migrants (N = 1,058). The group of non-migrants was matched to the group of migrants based on gender, age and educational level. Migrants were overrepresented in the study sample because the questionnaire was also used for other studies, which specifically focused on migrant groups. One postal reminder (after two weeks), and two electronic reminders (after one and after two weeks) were sent to panel members who did not respond. Panel members were free to answer the questions or not. The questionnaire was returned by 974 panel members (response rate 46%).

### Measurements

#### Involvement in medical decision-making

We used two items to measure involvement in medical decision-making. 1) How often do you think that your doctor takes the decisions about what’s best for your health? And, 2) how often do you think that the important medical decisions will be taken by your doctor and not by yourself? The options for answering were: 1) never; 2) sometimes; 3) often, and; 4) always. Both items were based on items developed by Flynn et al. (2006) [40]. An earlier study, using the same two items as dependent variable, showed that both measured a single concept (Cronbach’s alpha 0.78) [41]. We recoded both items (1 = 4, 2 = 3 etc.), and only included respondents that filled out both (included: N = 956, excluded: N = 18). A mean score was calculated ranging from 1 to 4, in which higher scores indicated that respondents report being more involved in medical decision-making. This mean score was, to a fair degree, normally distributed.

#### Health literacy

We used five scales of the Health Literacy Questionnaire (HLQ) developed by Osborne et al. (2013) [42] in order to measure the concept of health literacy. The complete HLQ includes nine different scales of health literacy skills based on 44 different items. As argued, competences such as finding and having enough information, understanding and appraising this information, and being able to engage with physicians are particularly relevant in the context of medical decision-making. We, therefore, included the following five scales of the HLQ: 1) having sufficient information to manage my health (four items); 2) appraisal of health information (five items); 3) ability to actively engage with health care providers (five items); 4) ability to find good health information (five items), and; 5) understanding health information well enough to know what to do (five items). In line with Nutbeam’s definition of health literacy, the scales one, four and five can be considered as functional literacy, scale three as communicative or interactive literacy, and scale two as critical literacy. For the items of the scales one and two, respondents were asked to what extent they agree with a statement, for example, ‘I have enough information to help me deal with my health problems’ and ‘I always compare health information from different sources and decide what is best for me’. The items could be answered using the following options: 1) strongly disagree; 2) disagree; 3) agree, and; 4) strongly agree. For the items of the scales three, four and five, respondents were asked how difficult or easy a number of tasks are for them. For example, tasks included: ‘Have good discussions about your health with doctors’, and; ‘Read and understand written health information’. These items could be answered using the following options: 1) cannot do; 2) very difficult; 3) quite difficult; 4) quite easy, and; 5) very easy. We used the Dutch version of the HLQ for our study. This has been translated and validated. We constructed a scale score for each of the five scales included instead of one total health literacy score. This is because we expected that the different health competences might have a different impact upon self-reported involvement. To construct these scale scores, mean scores were calculated for the five scales included for each respondent. If responses to one or two items were missing, the mean of the available items was used. If more than two items in a scale were missing, the scale score was regarded as missing. The number of respondents excluded per scale ranged from N = 48 to N = 96. The internal consistency given by Cronbach’s alpha varied, depending on the scale, between 0.73 and 0.89. The mean scores for the scales having sufficient information to manage my health and appraisal of health information ranged from 1 to 4, whereas the mean scores for the scales ability to actively engage with health care providers, ability to find good health information, and understanding health information well enough to know what to do ranged from 1 to 5. For all scales, higher scores indicated higher health literacy.

#### Socio-demographics

The following socio-demographics were included: age (continuous); gender (0 = man, 1 = woman); highest level of education completed (1 = low, 2 = middle, and 3 = high); ethnicity (1 = non-migrant, 2 = western migrant, and 3 = non-western migrant), and; self-reported general health (1 = excellent/very good, 2 = good, and 3 = fair/bad).

### Statistical analyses

We performed descriptive statistics first in order to describe the characteristics of the respondents. Secondly, we constructed a multiple linear regression model (model-I) including self-reported involvement as the dependent variable, and the socio-demographics as the independent variables, in order to test whether our data are consistent with earlier studies. Thirdly, we tested the association between health literacy and involvement in medical decision-making. We ran five different multiple linear regression analyses. In each analysis, we included self-reported involvement as a dependent variable, and one of the five health literacy scales and the socio-demographics as independent variables (model-II to model-VI). We did not include all five scales in one model because of the correlations between the scales (0.21 to 0.84). We controlled all models for whether a respondent filled out the questionnaire through the internet (1), or by post (0). In the regression analyses, categorical variables (educational level, ethnicity and self-reported general health) were recoded into dummy variables. The level of statistical significance was fixed at 0.05. All statistical analyses were carried out using STATA, version 13.1.

## Results

Approximately half (53%) of the respondents were women and the mean age of the respondents was 63 years (range 19 to 90 years; Table 1). About half (51%) had a middle level of education. Table 1 shows that 55% of the respondents were non-migrant, 36% western migrant and 9% non-western migrant. General health was self-reported as excellent/very good by 28% of the respondents. Compared to the general Dutch population aged 18 years and older, there was some overrepresentation in the group of respondents, mainly in elderly (65 years and older) and western migrants [37]. The reason for this is that these groups were also overrepresented in the study sample due to the fact that the questionnaire was also used for other studies focusing on migrant groups.

**Table 1:** Descriptive statistics of the respondents.

	N		% or mean (SD)
**Gender**	**974**		
Male		461	47.3
Female		513	52.7
**Age**	**974**		63 (15.7)
**Level of education**	**955**		
Low (none, primary school or pre-vocational education)		154	16.1
Middle (secondary or vocational education)		485	50.8
High (professional higher education or university)		316	33.1
**Ethnicity**	**974**		
Non-migrant		532	54.6
Western migrant		353	36.2
Non-western migrant		89	9.1
**Self-reported general health**	**925**		
Excellent/very good		257	27.8
Good		454	49.1
Fair/bad		214	23.1
**Questionnaire**	**974**		
Post		499	51.2
Internet		475	48.8
**Involvement in medical decision-making** (range 1–4, higher scores indicate more involvement)	**956**		2.40 (0.78)
**HLQ** (higher scores indicate higher levels of health literacy)			
Having sufficient information to manage my health (range 1–4)	**926**		2.85 (0.39)
Appraisal of health information (range 1–4)	**919**		2.62 (0.45)
Ability to actively engage with health care providers (range 1–5)	**892**		3.82 (0.62)
Ability to find good health information (range 1–5)	**891**		3.80 (0.61)
Understanding health information well enough to know what to do (range 1–5)	**878**		3.89 (0.57)

The mean score for self-reported involvement in medical decision-making was 2.40 (SD 0.78) on a scale from 1 (no involvement) to 4 (most involvement) (Table 1). Approximately half (49%) of the respondents had a score of 2 or lower. This means that, on average, respondents were slightly more inclined to leave medical decision-making to the physician. Table 1 also presents the mean scores per health literacy scale.

Younger people (β = -0.102, p = 0.004) and women (β = 0.121, p = 0.000) report being more involved in medical decision-making than older people and men (Table 2, model-I). Also, people with a high level of education (β = 0.240, p = 0.000) report being more involved in medical decision-making compared with people with a low level of education. No significant association was observed for ethnicity and self-reported general health. The explained variance of the model was 7%.

**Table 2:** Regression models to examine the association between health literacy and self-reported involvement in medical decision-making.

	Influence socio-demographics on self-reported involvement (MODEL-I) (N = 897)			Influence having sufficient information to manage my health on self-reported involvement (MODEL-II) (N = 888)			Influence appraisal of health information on self-reported involvement (MODEL-III) (N = 882)			Influence ability to actively engage with health care providers on self-reported involvement (MODEL-IV) (N = 855)			Influence ability to find good health information on self-reported involvement (MODEL-V) (N = 855)			Influence understand health information well enough to know what to do on self-reported involvement (MODEL-VI) (N = 842)		
Self-reported involvement (range 1–4, higher scores indicate more involvement)	Coef.	Beta^a^	P-value	Coef.	Beta ^a^	P-value	Coef.	Beta ^a^	P-value	Coef.	Beta ^a^	P-value	Coef.	Beta ^a^	P-value	Coef.	Beta ^a^	P-value
**Gender** (0 = man; 1 = woman)	0.188	0.121	**0.000**	0.192	0.123	**0.000**	0.179	0.115	**0.001**	0.222	0.143	**0.000**	0.215	0.139	**0.000**	0.219	0.140	**0.000**
**Age** (continuous)	-0.005	-0.102	**0.004**	-0.005	-0.102	**0.004**	-0.005	-0.101	**0.004**	-0.005	-0.107	**0.003**	-0.005	-0.103	**0.005**	-0.005	-0.106	**0.004**
**Level of education**																		
Low	Ref	Ref	Ref	Ref	Ref	Ref	Ref	Ref	Ref	Ref	Ref	Ref	Ref	Ref	Ref	Ref	Ref	Ref
Middle	0.112	0.072	0.123	0.112	0.072	0.127	0.112	0.072	0.127	0.115	0.074	0.124	0.089	0.057	0.237	0.104	0.067	0.168
High	0.394	0.240	**0.000**	0.390	0.237	**0.000**	0.375	0.228	**0.000**	0.396	0.241	**0.000**	0.355	0.215	**0.000**	0.370	0.223	**0.000**
**Ethnicity**																		
Non-migrant	Ref	Ref	Ref	Ref	Ref	Ref	Ref	Ref	Ref	Ref	Ref	Ref	Ref	Ref	Ref	Ref	Ref	Ref
Western migrant	-0.008	-0.005	0.879	-0.008	-0.005	0.883	-0.013	-0.008	0.815	0.015	0.009	0.786	0.003	0.002	0.956	0.001	0.001	0.981
Non-western migrant	-0.025	-0.009	0.790	-0.031	-0.011	0.744	-0.057	-0.020	0.552	-0.093	-0.033	0.350	-0.078	-0.028	0.431	-0.022	-0.008	0.829
**Self-reported general health**																		
Excellent/very good	Ref	Ref	Ref	Ref	Ref	Ref	Ref	Ref	Ref	Ref	Ref	Ref	Ref	Ref	Ref	Ref	Ref	Ref
Good	0.050	0.032	0.412	0.046	0.029	0.457	0.051	0.033	0.406	0.025	0.016	0.691	0.033	0.021	0.600	0.008	0.005	0.895
Fair/bad	0.055	0.030	0.452	0.061	0.033	0.422	0.055	0.030	0.457	0.015	0.008	0.850	0.052	0.028	0.507	0.025	0.013	0.751
**Questionnaire** (0 = post; 1 = internet)	0.140	0.090	**0.009**	0.136	0.087	**0.012**	0.126	0.081	**0.020**	0.140	0.090	**0.011**	0.136	0.088	**0.013**	0.145	0.093	**0.009**
**Having sufficient information to manage my health** (range 1–4)				0.042	0.021	0.527												
**Appraisal of health information** (range 1–4)							0.187	0.109	**0.001**									
**Ability to actively engage with health care providers** (range 1–5)										-0.067	-0.053	0.132						
**Ability to find good health information** (range 1–5)													0.033	0.025	0.487			
**Understand health information well enough to know what to do** (range 1–5)																0.017	0.012	0.741
**Constant**	2.338	-	**0.000**	2.217	-	**0.000**	1.864	-	**0.000**	2.609	-	**0.000**	2.238	-	**0.000**	2.312	-	**0.000**
**Adjusted R-square**		0.070			0.069			0.079			0.071			0.069			0.072	

^**a**^ *Standardized coefficient*.

We added the health literacy scales as independent variables in the models-II to VI in order to examine whether health literacy might be an explanation for the variation observed in the involvement in medical decision-making. Table 2 showed that it is only the health literacy scale appraisal of health information (model-III) which is associated significantly with self-reported involvement in medical decision-making (β = 0.109, p = 0.001). The higher respondents scored on the scale the appraisal of health information, the more they reported being involved in medical decision-making. Just as in model-I, women (β = 0.115, p = 0.001), younger people (β = -0.101, p = 0.004) and high educated people (β = 0.228, p = 0.000), report being more involved in medical decision-making. The explained variance of model-III was 8%. No significant association with self-reported involvement was observed for the other four health literacy scales. We did find significant associations in all these four models between the socio-demographics gender, age and educational level and self-reported involvement in medical decision-making (Table 2).

## Discussion

This study sought to gain insight into the relationship between health literacy and self-reported involvement in medical decision-making. We focused on a broad concept of health literacy instead of only on functional health literacy. In general, our results did not show a positive association between health literacy and self-reported involvement. We found that the higher respondents score on the health literacy scale appraisal of health information, the more they report being involved. Our hypothesis is, therefore, only partly confirmed.

We did not find a relationship between most aspects of health literacy and involvement in medical decision-making. Previous research generally found a positive relationship between health literacy and involvement [30–36]. It is difficult to compare our results with these previous studies as these predominantly focused on the relationship between functional health literacy, for example simply reading health information, and peoples’ preferences towards participation. Our study focused on a broader concept of health literacy and on self-reported involvement in medical decision-making. As such, we included a range of various resources and skills covering all domains of health literacy instead of only including reading skills. Furthermore, these studies were mainly performed among specific patient groups. We performed our study among a general sample of health care users in the Netherlands.

We observed a positive significant relationship between the health literacy scale appraisal of health information and self-reported involvement in medical decision-making. This suggests that higher order competences, that is critical health literacy, are more important when it comes to reporting involvement in medical decision-making as opposed to functional and communicative or interactive competences. Chinn (2011) noticed that “information appraisal” is one component of critical health literacy. Critical appraisal of information is about cognitive skills in managing and interpreting information, as well as about assessing the personal relevance of information [43]. In the context of our study, it appears that in order to take an active role in the decision-making process people have to be able to interpret information and weigh this information against their own preferences.

It has been argued that both functional and communicative or interactive health literacy need to be in place in order for critical health literacy to emerge [44]. Our results do not support this, as we only observed a relationship between critical health literacy and involvement, and not between functional and communicative or interactive health literacy and involvement. One possible explanation for this might be that our respondents in general scored quite high on these lower order competences. It has also been suggested that the functional, communicative or interactive and critical components of health literacy can be seen as complementary [45]. Research showed that it depends upon the type of behavior which health literacy skills are required. Different types of health literacy, be they functional, communicative or interactive, or critical, have a different impact upon different outcome measures [45 46]. Our results are in line with this. This means that it is possible that when other outcome measures such as preferences, or wishes, for involvement are used, a relationship might be found between other types of health literacy and these outcomes. Future research is recommended to unravel further the relationship between health literacy and involvement. This may enable researchers to gain more insight into whether health literacy might be an asset to enhance patient participation in medical decision-making.

Besides the influence of the health literacy scale appraisal of health information, we also found that younger people, women, and highly educated people report being more involved in the decision-making process. These results are consistent with previous empirical research showing that involvement in medical decision-making is associated with socio-demographics [10 12 13].

Our model-III does not explain much of the variance in self-reported involvement in medical decision-making. This implies that factors other than health literacy and socio-demographics explain such involvement. One of these mechanisms is someone’s social context, as this influences individual behavior [47]. Someone’s social network affects individual behavior through different mechanisms, such as social support and social norms [48–50]. Another factor that might play a role is the trust that patients have in physicians.

A first strength of this study is the use of the health literacy questionnaire (HLQ). This HLQ is a comprehensive measurement of health literacy encompassing both a broad range of resources and various skills [42]. Previous studies investigating the relationship between health literacy and involvement in medical decision-making included only functional health literacy by using, for instance, the Test of Functional Health Literacy in Adults (TOFHLA). Although focusing on a narrow conception of health literacy, instruments like the TOFHLA have the virtue of directly assessing peoples capabilities. A limitation of the HLQ is that it is a subjective measurement; it provides an indication of the perceived resources and skills of people. However, currently, no universally agreed measurement which captures all aspects of the concept of health literacy is available [51]. Another strength is the large sample size, even though the response rate was less than 50%. However, a limitation is that there are several challenges to how far our results can be applied generally. Our respondents were not representative of the general Dutch population aged 18 years and older. By comparison with this population, mainly elderly (65 years and older) and western migrants were overrepresented in this study’s group of respondents [37]. The reason for this is that these groups were also overrepresented in the study sample due to the fact that the questionnaire was also used for other studies focusing on migrant groups. We expect, however, that this does not affect our regression results, since all subgroups are of sufficient size for association analyses. It can also be argued that members of a health care panel are more interested in health care and therefore might take a more active role in medical decision-making. Furthermore, it is possible that we overestimate the level of health literacy in our sample due to the nature of the data collection—as people with very low levels of health literacy may not participate in a written questionnaire. Our results show that, except for the appraisal of health information, respondents score quite high on the scales included. Another limitation might be that we examined self-reported involvement in medical decision-making, instead of actual observed behavior. In the questionnaire, we did not refer to a specific treatment decision, but asked about involvement in general. It remains unclear from this study whether people do indeed take an active role in a situation where a specific, concrete decision is made. From the literature it is known that the type of care people have to decide upon has an impact upon the importance they attach to shared decision-making, as well as upon their actual involvement in decision-making [52]. Using self-reported involvement as an outcome might also be a strength, as this gives insight into how people themselves experience their involvement. We also examined self-reported levels of health literacy. In a real life situation, people might have more difficulty with health literacy than they reported. For example due to emotions such as stress. In addition, it is possible that respondents with lower capabilities overestimate their abilities, whereas respondents with higher capabilities tend to underestimate their abilities [53]. A final limitation is that our data were obtained using a cross-sectional study design, and as such cannot provide any information about causal relationships.

## Conclusion

This study suggests that higher order competences—that is critical health literacy—in particular are important for the reporting of involvement in medical decision-making. Future research is recommended to unravel further the relationship between health literacy and patient involvement in order to gain insight into whether health literacy might be an asset to enhance patient participation in medical decision-making.
